# Prenatal genetic findings and pregnancy outcomes in fetuses with congenital heart disease: a decade-long retrospective analysis at a tertiary hospital

**DOI:** 10.3389/fmed.2026.1870270

**Published:** 2026-08-17

**Authors:** Qingcheng Chen, Longzhuang Peng, Youchun Cai, Xiuqing Qiu, Qiumei Wu, Guorong Lyu, Fengying Ye, Wen Ling

**Affiliations:** 1 Department of Medical Ultrasonics, Ningde Clinical Medical College of Fujian Medical University, Ningde, Fujian, China; 2 Department of Medical Ultrasonics, Ningde Municipal Hospital, Ningde, Fujian, China; 3 Department of Medical Ultrasonics, Fujian Maternity and Child Health Hospital, College of Clinical Medicine for Obstetrics & Gynecology and Pediatrics, Fujian Medical University, Fuzhou, Fujian, China; 4 Department of Obstetrics and Gynecology, Fujian Maternity and Child Health Hospital, College of Clinical Medicine for Obstetrics & Gynecology and Pediatrics, Fujian Medical University, Fuzhou, Fujian, China; 5 Department of Medical Ultrasonics, the Second Affiliated Hospital of Fujian Medical University, Quanzhou, Fujian, China

**Keywords:** congenital heart disease, genetic abnormalities, pregnancy outcome, prenatal diagnosis, ultrasound phenotype

## Abstract

**Objective:**

This study aimed to investigate the relationship between fetal congenital heart disease (CHD) phenotypes and genetic abnormalities and to evaluate the use of stepwise genetic testing [karyotyping, chromosomal microarray analysis (CMA), and whole-exome sequencing (WES)] for prenatal diagnosis and outcome prediction.

**Methods:**

This retrospective study included 1,354 CHD fetuses undergoing karyotyping and CMA; cases with negative results underwent WES. CHD was classified by type, complexity, and extracardiac anomalies. Phenotype-outcome associations were analyzed.

**Results:**

Among 1,354 fetuses, karyotyping and CMA detected pathogenic abnormalities in 11.5% (156/1.354); WES identified 16 pathogenic/likely pathogenic (P/LP) (12.2%) and 2 variant of uncertain significance (VOUS) (1.5%) variants among the 131 cases negative on karyotyping and CMA. The highest detection rates occurred in conotruncal anomalies (25.0%) and atrioventricular septal defects (45.5%). Rates were higher in non-isolated versus isolated CHD (*p* < 0.001) and complex versus simple CHD (*p* < 0.05). Pathogenic abnormalities were associated with lower live birth rates (*p* < 0.001), largely attributed to pregnancy termination.

**Conclusion:**

Fetal CHD is strongly associated with genetic abnormalities, which can be effectively detected via stepwise testing (karyotyping, CMA, and WES) and are linked to adverse outcomes. Ultrasound findings suggest that genetic risk varies by CHD type, complexity, and extracardiac anomalies. Integrating detailed phenotypes may inform more precise prenatal genetic risk stratification, though prospective validation is needed to establish clinical utility.

## Introduction

Congenital heart disease (CHD), resulting from embryonic developmental anomalies of the heart and great vessels, is the most common birth defect, affecting 0.9% of live births and accounting for 30–50% of infant deaths (1, 2).

Ultrasound is the primary method for diagnosis, with 81–97% accuracy in diagnosing CHD (3). CHD etiology is multifactorial, with genetic factors accounting for a substantial proportion of cases (40–60%) (4–7). Chromosomal abnormalities, the most common genetic cause, account for 20% of cases, including numerical and structural anomalies, and are often associated with complex defects such as atrioventricular septal defect (AVSD) and tetralogy of Fallot (TOF) (8). At the genomic level, copy number variants (CNVs) and single nucleotide variants (SNVs) are also involved. Even with normal chromosome numbers and CNVs, 3–5% of CHD fetuses carry SNVs. Patients with genetic anomalies generally have a poorer prognosis (1); thus, genetic testing is recommended when fetal CHD is detected (6, 9).

Current prenatal genetic testing strategies have limitations. Traditional serum screening, while convenient, has a limited detection rate and low positive predictive value due to its reliance on biochemical and demographic markers. Non-invasive prenatal testing demonstrates high sensitivity for common aneuploidies. However, as a screening tool targeting specific chromosomal abnormalities, it is not designed to detect the full spectrum of CHD-associated genetic etiologies, which predominantly involve structural chromosomal aberrations and single-gene variants requiring diagnostic testing such as chromosomal microarray analysis (CMA) and whole-exome sequencing (WES) (10).

Invasive prenatal diagnosis (amniocentesis, cordocentesis, or chorionic villus sampling) with karyotyping remains the conventional approach to detecting fetal chromosomal abnormalities. This technique provides direct visualization of numerical anomalies and large structural aberrations (e.g., balanced translocations), but it requires cell culture, has a long turnaround time, offers low resolution (>5 Mb), and cannot detect microdeletions/duplications.

CMA overcomes some limitations by simultaneously detecting aneuploidies and CNVs at a genome-wide scale. Two main platforms exist: array comparative genomic hybridization, which detects CNVs, and single nucleotide polymorphism array (SNP array), which additionally identifies uniparental disomy and triploidy (11). CMA has been recommended by professional societies as the first-tier cytogenetic test for prenatal diagnosis of fetuses with structural anomalies, including CHD, for over a decade. While CMA offers a significantly higher diagnostic yield for CHD-related abnormalities than conventional karyotyping, it cannot detect balanced translocations, inversions, or low-level mosaicism—scenarios in which karyotyping may retain a supplementary role (12). Nevertheless, karyotyping is no longer considered the primary diagnostic approach in current prenatal CHD evaluation.

WES provides ultra-high resolution by deep sequencing of 20,000 protein-coding genes with a shorter turnaround time. Studies show that WES identifies pathogenic single-gene variants in an additional 10% of CHD fetuses with normal karyotype and CMA results (13, 14). The American College of Medical Genetics and Genomics (ACMG) evidence-based guideline strongly recommends that exome or genome sequencing be considered as a first- or second-tier test for patients with congenital anomalies, such as CHD, and recent ACMG guidance further indicates that CMA is no longer the sole first-tier genetic test. While WES has expanded diagnostic capabilities, sequential testing starting with CMA may remain a practical strategy in certain prenatal settings, particularly where access to WES is limited.

Current CHD research focuses primarily on genetic etiology identification, but systematic analysis linking specific prenatal ultrasound phenotypes to genetic abnormalities—and their distribution across CHD types—is lacking. This study aims to investigate the association between fetal CHD phenotypes and genetic anomalies and to preliminarily elucidate the underlying genetic etiology. Moreover, by integrating genetic findings with pregnancy outcomes, we aimed to provide evidence for prenatal counseling, prognosis, and clinical decision-making.

## Materials and methods

### Patients

This retrospective study included fetuses diagnosed with CHD by ultrasound at Fujian Maternity and Child Health Hospital from January 2014 to December 2023. Inclusion criteria were as follows: (1) prenatal ultrasound indicating fetal CHD, excluding isolated soft markers (e.g., cardiac echogenic foci), benign variants without structural anomalies (e.g., isolated atrial septal aneurysm), and functional abnormalities without structural defects (e.g., isolated arrhythmias or valvular regurgitation); (2) both karyotyping and CMA performed prenatally as the initial testing strategy, with cases negative for both undergoing WES when parental consent and adequate DNA samples were available (reflecting the sequential testing approach used during the study period, 2014–2023); and (3) for live births, postnatal transthoracic echocardiography was performed at our hospital to confirm prenatal diagnosis, with follow-up to 3–6 months to record growth and development. Written informed consent for invasive prenatal testing and subsequent genetic analysis was obtained from all pregnant women prior to enrollment. Consent for the use of anonymized clinical data in this retrospective study was covered under the ethical approval granted by the Institutional Review Board. This study was approved by the Institutional Review Board of Fujian Maternity and Child Health Hospital (Approval No. 2024KY129).

### Ultrasound and genetic testing

Ultrasound was performed by certified physicians using GE Voluson E8/E10/S8, PHILIPS EPIQ ELITE/EPIQ7, and WS80A systems with 2D convex (2–9 MHz), 3D volume (4–8 MHz), and endocavitary (5–9 MHz) probes. All CHD diagnoses were confirmed by two physicians (with at least one senior physician) through consensus.

Sample collection: Fetal samples were obtained via ultrasound-guided amniocentesis (20 mL for karyotyping, 5–10 mL for SNP array) or cordocentesis (2.5 mL each for karyotyping and SNP array).

Karyotyping: Samples were cultured, G-banded, and analyzed using an automatic chromosome analyzer (PerkinElmer, USA). Thirty metaphases were counted, and five metaphases were analyzed; in abnormal cases, the results were confirmed by analyzing additional metaphases. Karyotypes were described according to the International System for Human Cytogenetic Nomenclature.

CMA: DNA was extracted using the QIAamp DNA Blood Mini Kit (Qiagen, Germany). CMA was performed with the Affymetrix CytoScan 750 K array according to the manufacturer’s protocol. CNVs were analyzed using Chromosome Analysis Suite software, with reporting thresholds of 200 kb (losses) and 500 kb (gains). CNVs were classified as pathogenic (P), likely pathogenic (LP), variant of uncertain significance (VOUS), likely benign (LB), or benign according to ACMG guidelines (15).

WES: Genomic DNA was extracted from fetal samples using the Tguide S32 system (Tiangen, China). Libraries were prepared with the KAPA HyperPlus Kit (Roche, Switzerland) and captured using the xGen Exome Research Panel v1.0 (IDT, USA). Sequencing was performed on the MGISEQ-2000 platform (MGI, China) with 150 bp paired-end reads. Reads were aligned to GRCh37/hg19 using Sentieon-BWA. Variant calling was performed with GATK, and annotations were added using Annovar. Pathogenicity was classified according to ACMG guidelines (16). WES was performed on probands only; parental samples were not included for trio analysis.

In this study, pathogenic genetic abnormalities were defined as chromosomal numerical anomalies detected by karyotyping or CMA, and P/LP CNVs or P/LP variants identified by CMA and WES, respectively. Of note, in this study, the term CNV refers specifically to submicroscopic deletions and duplications detected by CMA, excluding whole-chromosome aneuploidies, which are reported separately as numerical chromosomal abnormalities.

### CHD anatomical classification and grouping

CHD was classified into seven anatomical categories: (I) septal defects, (II) conotruncal anomalies, (III) right heart malformations, (IV) left heart malformations, (V) venous drainage anomalies, (VI) cardiac position abnormalities, and (VII) other cardiac anomalies.

Based on defect complexity and the presence of extracardiac anomalies, cases were divided into four groups: Group A (simple CHD without extracardiac anomalies), Group B (complex CHD without extracardiac anomalies), Group C (simple CHD with extracardiac anomalies), and Group D (complex CHD with extracardiac anomalies). Simple CHD was defined as the presence of intracardiac lesions without significant hemodynamic changes, such as an isolated ventricular septal defect (VSD). Complex CHD was defined as the presence of one or more concurrent cardiac or great vessel malformations with severe hemodynamic implications, such as TOF. Cases with multiple complex anomalies were classified by the primary defect; multiple simple defects did not constitute complex CHD.

### Statistical analysis

Categorical variables were presented as frequencies and percentages. Comparisons between groups were performed using the chi-square test or Fisher’s exact test, as appropriate. *Post hoc* pairwise comparisons were conducted using the chi-square partition method, with *p*-values adjusted by Bonferroni correction.

All statistical analyses were performed using SPSS version 19.0. All tests were two-sided, and statistical significance was set at *α* = 0.05; a *p*-value of <0.05 was considered statistically significant.

## Results

### Clinical characteristics

**Figure 1:**
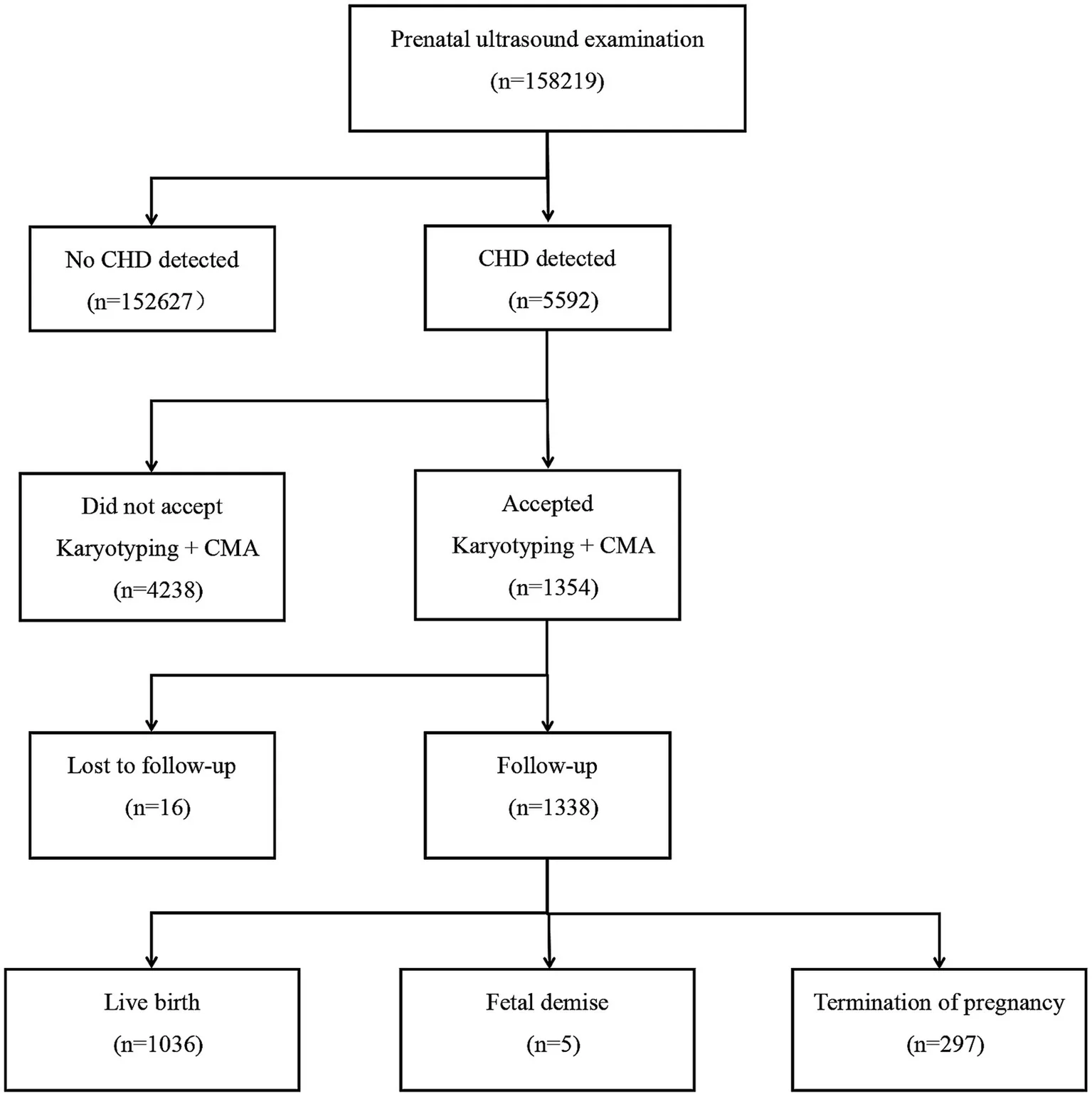
Flowchart of inclusion criteria.

A total of 1,354 fetuses with ultrasound-detected CHD who underwent both karyotyping and CMA were included in this study (Figure 1). The gestational age at CHD detection ranged from 11 + 2 to 38 + 1 weeks (mean, 24 + 2 weeks). Among these, 73 cases were detected in the first trimester (11~13 + 6 weeks), 1,013 cases in the second trimester (14~27 + 6 weeks), and 268 cases in the third trimester (≥28 weeks).

### Karyotyping results

Among the 1,354 fetuses with CHD, chromosomal abnormalities were detected by karyotyping in 124 cases (9.2%), including 82 numerical abnormalities (66.1%) and 42 structural abnormalities (33.9%) (Table 1). Among the 25 fetuses with balanced structural rearrangements (4 translocations and 21 inversions), pregnancy outcomes were as follows: 18 resulted in live births, 6 in pregnancy termination, and 1 was lost to follow-up.

**Table 1:** Results of abnormal karyotyping in 124 cases.

Category	Number	Detection rate of abnormal karyotype (%)	Proportion of abnormal karyotypes (%)
Numerical chromosomal abnormalities	82	6.1	66.1
Trisomy 21	32	2.4	25.8
Trisomy 18	24	1.8	19.4
Trisomy 13	4	0.3	3.2
Sex chromosome numerical abnormalities	17	1.3	13.7
Mosaic numerical chromosomal abnormalities	5	0.4	4.0
Structural chromosomal abnormalities	42	3.1	33.9
Unbalanced structural abnormalities	17	1.3	13.7
Translocation	4	0.3	3.2
Inversion	21	1.6	16.9

One-case discrepancy in numerical abnormality counts exists between Tables 1, 2; see the Discussion section for details.

### CMA results

Among the 1,354 fetuses with CHD, CMA detected abnormalities in 184 cases (13.6%), including 81 numerical abnormalities (44.0%), 64 P CNVs (34.8%), 11 LP CNVs (6.0%), 27 VOUS CNVs (14.7%), and 1 LB CNV (0.5%) (Table 2). The VOUS and LB CNVs were not classified as pathogenic abnormalities.

**Table 2:** Results of abnormal CMA in 184 cases.

Category	Number	Detection rate of abnormal CMA (%)	Proportion of abnormal CMA (%)
Numerical chromosomal abnormalities	81	6.0	44.0
Trisomy 21	32	2.4	17.4
Trisomy 18	22	1.6	12.0
Trisomy 13	4	0.3	2.2
Tetrasomy 18	1	0.1	0.5
Sex chromosome numerical abnormalities	17	1.3	9.2
Mosaic numerical chromosomal abnormalities	5	0.4	2.7
P CNVs	64	4.7	34.8
LP CNVs	11	0.8	6.0
VOUS CNVs	27	2.0	14.7
LB CNVs	1	0.1	0.5

One-case discrepancy in numerical abnormality counts exists between Tables 1 and 2; see the Discussion section for details.

Of the 75 P/LP submicroscopic CNVs identified, 42 had an established or likely causal relationship with CHD, including 27 previously reported CNVs (Supplementary Table 1, No. 1–21) and 15 less frequently reported CNVs (Supplementary Table 1, No. 22–42), while the remaining 33 showed no clear association with CHD.

Of the 27 previously reported P/LP CNVs (Supplementary Table 1, No. 1–21), a 22q11.2 microdeletion/duplication was identified in 21 cases (involving the critical gene *TBX1*). The main ultrasound phenotypes included VSD (*n* = 5), aberrant right subclavian artery (*n* = 4), TOF (*n* = 3), pulmonary atresia (*n* = 2), truncus arteriosus (*n* = 1), and interrupted aortic arch (*n* = 1). Two cases of 17p11.2 microdeletion/duplication (involving *RAI1*) presented with VSD and right aortic arch. One case each presented as follows: 7q11.23 microdeletion (Williams–Beuren syndrome, involving *ELN*) with VSD; 5p15.33 microdeletion (cri-du-chat syndrome region) with truncus arteriosus; 4p16.3 deletion (involving *WHSC1* and *FGFRL1*) with aortic stenosis; and 11q23.3 microdeletion (Jacobsen syndrome region) with VSD.

Of the 15 less reported P/LP CNVs (Supplementary Table 1, No. 22–42), 15q11.2 deletion was identified in 6 cases [involving the BP1-BP2 (breakpoint 1 to breakpoint 2) region], with primary ultrasound phenotypes including VSD (*n* = 2), aberrant right subclavian artery (*n* = 2), dextrocardia (*n* = 1), and coarctation of the aorta (*n* = 1). Four cases of 16p11.2 microdeletion/duplication (involving *HIRIP3*) presented with TOF (*n* = 3) and aberrant right subclavian artery (*n* = 1). Two cases of 1p36.33 microdeletion (involving *SKI*) presented with ventricular septal defect and ductus venosus-systemic venous shunt. One case each presented as follows: 1q21.1 microdeletion/duplication (involving *GJA5*, *PRKAB2*, and *CHD1L*) with an aberrant right subclavian artery; 8q21.11 microdeletion (involving *HEY1*) with VSD and cleft palate; and 15q24.1q24.2 microdeletion (15q24 deletion syndrome region) with VSD.

Among the 33 submicroscopic P/LP CNVs without a clearly established causal relationship with CHD, examples included Xq28 deletion (*n* = 2, encompassing genes including *F8* and *RAB39B*), 4q22.2q24 deletion (*n* = 1, encompassing *GRID*), and 17p12 deletion (*n* = 1, encompassing *PMP22*).

Among the 27 VOUS CNVs, the main ultrasound phenotypes included VSD (*n* = 13), persistent left superior vena cava (PLSVC) (*n* = 4), and pulmonary stenosis or atresia (*n* = 3) (Supplementary Table 2).

The single LB CNV case was an 8q24.3 microduplication with ultrasound findings of VSD and PLSVC.

### WES results

Of the 1,198 fetuses with negative karyotyping and CMA results, 131 (10.9%) underwent WES. The remaining 1,067 cases did not undergo WES primarily due to logistical constraints, such as limited DNA availability and parental preference. Among these, WES identified variants in 18 cases (13.7% of the 131 tested), including 5 P variants and 11 LP variants (16/131, 12.2%), and 2 VOUS (2/131, 1.5%) (Supplementary Table 3). VOUS were not classified as pathogenic abnormalities.

These 18 variants involved 18 genes: *NONO*, *MYLK*, *ZEB2*, *KMT2D*, *EFEMP2*, *Tab2*, *DSP*, *TSC2*, *PKP2*, *KDR*, *TTC37*, *NODAL*, *TCAP*, *COL3A1*, *NEK1*, *TRPM4*, *SON*, and *NIPBL*. Of these, 17 were compound heterozygous variants, and 1 was a hemizygous variant. Eight genes were significantly associated with CHD ultrasound phenotypes (*NONO*, *MYLK*, *ZEB2*, *KMT2D*, *EFEMP2*, *Tab2*, *DSP*, and *TSC2*), and seven showed potential associations (*PKP2*, *KDR*, *TTC37*, *NODAL*, *TCAP*, *COL3A1*, and *NEK1*). The two VOUS involved variants in *TRPM4* and *SON*.

### Ultrasound findings

A total of 34 CHD types were identified. The ten most common were VSD (41.0%), vascular ring (29.2%), PLSVC (8.7%), coarctation of the aorta (5.0%), TOF (5.0%), pulmonary stenosis (1.6%), pulmonary atresia (1.0%), DORV (0.8%), AVSD (0.8%), and transposition of the great arteries (0.8%), accounting for 93.8% of all cases (Supplementary Table 4).

Based on anatomical classification, the 1,354 CHD cases were categorized as follows: (I) septal defects (*n* = 567), (II) conotruncal anomalies (*n* = 92), (III) right heart lesions (*n* = 49), (IV) left heart lesions (*n* = 89), (V) anomalous venous drainage (*n* = 123), (VI) cardiac malpositions (*n* = 21), and (VII) others (*n* = 413) (Supplementary Table 4).

Based on defect complexity and extracardiac anomalies, cases were divided into Group A (*n* = 904), Group B (*n* = 237), Group C (*n* = 131), and Group D (*n* = 82). Groups A and B comprised isolated CHD, while Groups C and D comprised non-isolated CHD.

Extracardiac anomalies were present in 213 fetuses (15.7%), with the most common being multisystem anomalies (27.2%), urogenital anomalies (14.6%), and central nervous system anomalies (12.6%), followed by digestive system anomalies, thoracic malformations, skeletal dysplasias, facial anomalies, hydrops syndrome, anterior abdominal wall defects, and other extracardiac anomalies.

### Pathogenic genetic abnormalities by CHD type

Among the 34 CHD types identified, the highest detection rates of pathogenic genetic abnormalities among relatively common CHD subtypes (*n* > 10) were observed in AVSD (5/11, 45.5%), pulmonary atresia (4/13, 30.8%), DORV (3/11, 27.3%), and TOF (17/67, 25.4%). Lower detection rates were found in PLSVC (5/118, 4.2%), VSD (50/555, 9.0%), and vascular ring (40/395, 10.1%). Additionally, several rare CHD subtypes (*n* ≤ 5) showed extremely high detection rates, including truncus arteriosus (3/3, 100%), HLHS (3/4, 75.0%), and absent pulmonary valve syndrome (1/1, 100%) (Supplementary Table 5). Notably, among the 40 cases of isolated muscular VSD identified in this study, no pathogenic genetic abnormalities were detected.

### Pathogenic genetic abnormalities by CHD anatomical classification

Among the 1,354 CHD cases, pathogenic genetic abnormalities were identified in 156 fetuses (11.5%), including 81 numerical abnormalities and 75 submicroscopic P/LP CNVs (Table 3).

**Table 3:** Comparison of detection rates of pathogenic genetic abnormalities by different anatomical classifications of CHD.

Anatomical classification	Number	Pathogenic genetic abnormalities		
		Total abnormal (%)	Numerical chromosomal abnormalities (%)	P/ LP submicroscopic CNVs (%)
VI. Cardiac position anomalies	21	1 (4.8)	0 (0)	1 (4.8)
V. Venous return anomalies	123	6 (4.9)	2 (1.6)	4 (3.3)
I. Septal defects	567	55 (9.7)	29(5.1)	26 (4.6)
VII. Other cardiac anomalies	413	45 (10.9)	24 (5.8)	21 (5.1)
III. Right heart anomalies	49	8 (16.3)	3 (6.1)	5 (10.2)
IV. Left heart anomalies	89	18 (20.2)	10 (11.2)	8 (9.0)
II. Conotruncal anomalies	92	23 (25.0)	13 (14.1)	10 (10.9)
Total	1,354	156	81	75

The highest detection rates of pathogenic genetic abnormalities were observed in Type II (25.0%), IV (20.2%), and III (16.3%). For numerical abnormalities, the highest rates were observed in Type II (14.1%), IV (11.2%), and III (6.1%). For P/LP CNVs, the highest rates were observed in Type II (10.9%), III (10.2%), and IV (9.0%) (Table 3).

The detection rate of pathogenic genetic abnormalities decreased in the following order: Type II > Type IV > Type III > Type VII > Type I > Type V > Type VI, with significant differences among groups (*p* < 0.001). Pairwise comparisons revealed significant differences between Type II and I, II and V, II and VII, and IV and V (all *p* < 0.002).

### Pathogenic genetic abnormalities by CHD grouping

Among the 1,354 CHD cases, pathogenic genetic abnormalities were detected in 90 of 1,141 isolated CHD cases (7.9%), including 39 numerical abnormalities and 51 submicroscopic P/LP CNVs. Among the 213 non-isolated CHD cases, 66 had pathogenic genetic abnormalities (31.0%), including 42 numerical abnormalities and 24 submicroscopic P/LP CNVs (Table 4).

**Table 4:** Detection of pathogenic genetic abnormalities in different ultrasonic groupings of CHD.

CHD group	Number (%)	Pathogenic genetic abnormalities		
		Total abnormal (%)	Numerical chromosomal abnormalities (%)	P/ LP submicroscopic CNVs (%)
Isolated CHD	1,141	90 (7.9)	39 (3.4)	51 (4.5)
Group A	904	58 (6.4)	26 (2.9)	32 (3.5)
Group B	237	32 (13.5)	13 (5.5)	19 (8.0)
Non-isolated CHD	213	66 (31.0)	42 (19.7)	24 (11.3)
Group C	131	36 (27.5)	20 (15.3)	16 (12.2)
Group D	82	30 (36.6)	22 (26.8)	8 (9.8)

The detection rates of pathogenic genetic abnormalities were significantly higher in non-isolated CHD (with extracardiac anomalies) than in isolated CHD (without extracardiac anomalies) (31.0% vs. 7.9%, *p* < 0.001).

The detection rate was significantly higher in complex CHD than in simple CHD, both in isolated cases (Group B vs. Group A: 13.5% vs. 6.4%, *p* < 0.05) and non-isolated cases (Group D vs. Group C: 36.6% vs. 27.5%, *p* < 0.05).

### WES findings and ultrasound phenotypes

Among the 131 CHD fetuses undergoing WES, 16 P/LP variants and 2 VOUS were identified. The ultrasound phenotypes of these fetuses are detailed in Supplementary Table 3.

**Figure 2:**
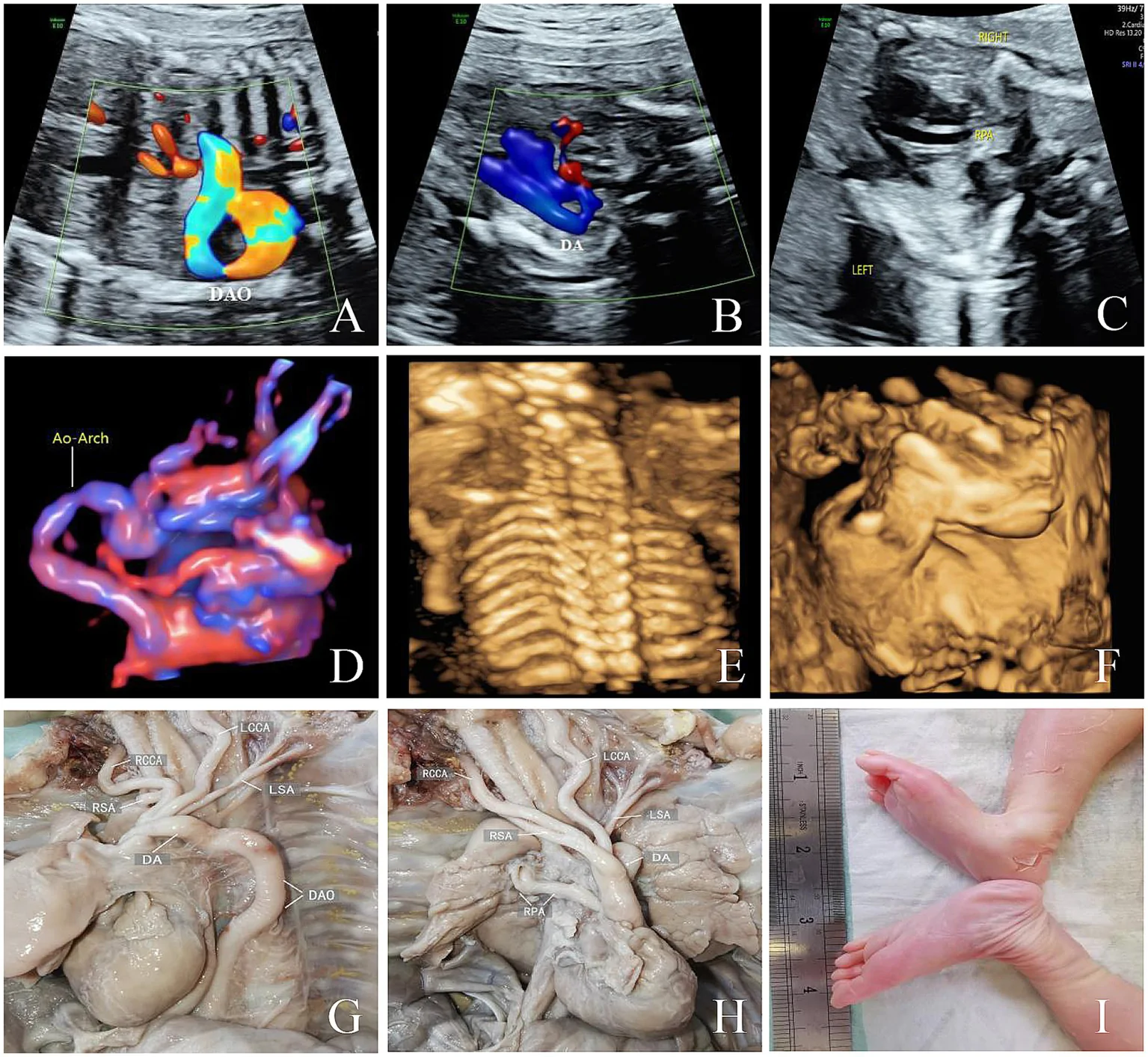
Ultrasound findings of *EFEMP2* gene variants. **(A)** Long-axis view of the aortic arch shows a tortuous descending aorta; **(B,C)** Three-vessel and tracheal views demonstrate elongation and tortuosity of the ascending aorta, ductus arteriosus, and bilateral pulmonary arteries; **(D)** 3D reconstruction shows tortuosity of the aortic arch and descending aorta; **(E)** Localized rib fracture; **(F)** Localized skin fold on the anterior chest; **(G–I)** Anterior view of anatomical specimens reveals tortuosity of the ascending aorta, aortic arch with its branches, and descending aorta, along with slender feet.

Eighteen variants across 18 genes were detected. Eight genes (*Tab2*, *NONO*, *DSP*, *KMT2D*, *MYLK*, *TSC2*, *EFEMP2*, and *ZEB2*) showed significant associations with CHD ultrasound phenotypes (Figure 2), while 10 genes (*TCAP*, *TTC37*, *COL3A1*, *PKP2*, *NODAL*, *KDR*, *NEK1*, *TRPM4*, *SON*, and *NIPBL*) showed potential associations (Supplementary Table 6).

### Pregnancy outcomes of CHD fetuses

Of the 1,354 CHD fetuses, 1,338 (98.8%) completed follow-up. Among these, 1,036 (77.4%) were live births, and 302 (22.6%) had adverse outcomes, including 5 intrauterine deaths and 297 terminations of pregnancy. Postnatal echocardiography confirmed prenatal diagnoses in five missed VSD cases, two missed pulmonary stenosis cases, two double aortic arches misdiagnosed as a right aortic arch, and one DORV misdiagnosed as transposition of the great arteries.

Pathogenic genetic abnormalities were identified in 172 fetuses (12.9%), while 1,166 (87.1%) had none. Among the 172 fetuses with pathogenic abnormalities, 155 resulted in termination, 1 in intrauterine death, and 16 in live births. Among the 1,166 fetuses without pathogenic abnormalities, 142 were terminated, 4 died in utero, and 1,020 were live births. The live birth rate was significantly higher in fetuses without pathogenic abnormalities (*p* < 0.001). Fetuses with pathogenic genetic abnormalities had a significantly higher rate of adverse pregnancy outcomes (156/172, 90.7%) compared with those without (146/1,166, 12.5%) (*p* < 0.001).

Among the 16 live births with pathogenic abnormalities, there were 11 P CNVs (5 with 15q11.2 deletion, 4 with 22q11.2 microdeletion/duplication, 1 with 17p12 duplication, 1 with Xq28 deletion), 3 LP CNVs (1 with 17p11.2 duplication, 1 with 1p36.21p35.2 duplication, 1 with 18q22.3q23 duplication), 1 P variant (*NONO*), and 1 LP variant (*TTC37*). Of these, 15 were term and 1 was preterm. Outcomes included the following: one in good condition post-surgery, one died at 1 month, four had language or developmental delay, one had intellectual disability, and the remainder were in good condition (Supplementary Table 7).

Although VOUS are not classified as pathogenic, we separately analyzed their outcomes for completeness. For the 19 live births with VOUS (18 detected by CMA, 1 by WES), 2 were lost to follow-up. Among the remaining 17 infants, 15 were term and 2 were preterm. Outcomes included the following: four infants were in good condition post-surgery, one died at 2 months, and the remainder were in good condition (Supplementary Table 8).

## Discussion

This study evaluated the relationship between CHD and genetic abnormalities. Among 1,354 cases, combined karyotyping and CMA identified pathogenic abnormalities in 11.5%. In cases with negative karyotyping and CMA results, WES detected P/LP variants in 12.2% and VOUS in 1.5%. Although current ACMG guidelines recommend exome or genome sequencing as a first- or second-tier test for congenital anomalies, our sequential approach—consistent with clinical practice during the study period (2014–2023)—offers an opportunity to evaluate the additional diagnostic yield of WES after conventional testing in a real-world prenatal setting. Genetic anomaly rates varied by CHD type, defect complexity, and the presence of extracardiac anomalies, underscoring the importance of detailed ultrasound phenotyping for personalized prenatal counseling. Furthermore, comprehensive follow-up of pregnancy outcomes clarified the prognosis of CHD fetuses with different ultrasound phenotypes and genetic profiles.

Before discussing these results, it is important to clarify our terminology. In this study, we used the term “pregnancy outcome” to refer to the final status of the pregnancy, namely, live birth, termination of pregnancy, or intrauterine fetal death. However, it is crucial to note that the overwhelming majority (297/302, 98.3%) of the adverse pregnancy outcomes in this cohort were terminations of pregnancy. Consequently, the observed association between pathogenic genetic abnormalities and lower live birth rates should be interpreted as primarily reflecting parental reproductive decisions made after prenatal genetic diagnosis rather than the direct biological lethality of the genetic abnormalities themselves. This distinction is critical for the interpretation of our findings and providing appropriate prenatal counseling.

Our findings suggest an association between fetal CHD ultrasound phenotypes and genetic risk. Initially, anatomical classification may serve as a reference for risk assessment. In this cohort, conotruncal anomalies (Type II), left heart malformations (Type IV), and right heart malformations (Type III) showed relatively high detection rates of pathogenic genetic abnormalities (25.0, 20.2, and 16.3%, respectively), consistent with previous studies reporting the highest genetic abnormality risk in conotruncal anomalies (17, 18). Therefore, for these ultrasound phenotypes, invasive prenatal diagnosis is warranted to rule out genetic abnormalities. It should be noted, however, that the high detection rate of pathogenic genetic abnormalities in conotruncal anomalies (25%, 23/92) was substantially driven by 22q11.2 microdeletions (*n* = 21), which is expected given the well-established role of TBX1 in outflow tract development. After excluding these deletions, pathogenic abnormalities were still detected in 2 of the remaining 71 cases (2.8%, 2/71), indicating that while 22q11.2 microdeletions are the predominant genetic cause in this anatomical group, other rare variants also contribute.

Furthermore, genetic risk varies across specific CHD types. The most common defects—VSD (41.0%), vascular ring (29.2%), and PLSVC (8.7%)—showed low detection rates of pathogenic abnormalities. Conversely, the less common TOF (5.0%) and AVSD (0.8%) had the highest rates (25.4 and 45.5%, respectively). Our TOF detection rate was consistent with that of Wang et al. (17) (25.4% vs. 25.7%), while our AVSD rate was lower (45.5% vs. 73.7%), likely due to a combination of factors, including a higher proportion of isolated AVSD without extracardiac anomalies in our cohort, differences in genetic testing strategies, and the relatively small sample size. As the most prevalent CHD type in this study, VSD exhibited no genetic abnormalities in isolated muscular cases, consistent with prior evidence indicating a lower genetic risk for isolated muscular VSD (19–22). However, given the limited sample size (*n* = 40) and the retrospective design, this finding is insufficient to justify withholding invasive testing; clinical decisions should continue to be guided by comprehensive ultrasound evaluation and individualized risk assessment.

Additionally, both defect complexity and extracardiac anomalies contributed to genetic risk in our cohort. The detection rate was higher in complex than in simple CHD in both isolated (13.5% vs. 6.4%, *P* < 0.05) and non-isolated cases (36.6% vs. 27.5%, *P* < 0.05), and even more markedly, in non-isolated than in isolated CHD (31.0% vs. 7.9%, *P* < 0.001). These findings, which extend previous inconsistent reports on defect complexity (19, 23, 24), underscore the importance of integrating both factors in genetic risk assessment.

CNVs represent an important molecular mechanism underlying fetal CHD (4, 25). In our cohort, 42 of the 75 submicroscopic P/LP CNVs were well-documented as being causally associated with CHD. The most frequently identified abnormality was the 22q11.2 microdeletion/duplication, which predominantly contributes to conotruncal anomalies, aortic arch abnormalities, and septal defects through the critical gene *TBX1* (26). Additional previously reported CHD-associated P/LP CNVs (27–29) identified in this study included 11q23.3 microdeletion (Jacobsen syndrome region), 5p15.33 microdeletion (cri-du-chat syndrome region), 7q11.23 microdeletion (*ELN*), 4p16.3 deletion (*WHSC1, FGFRL1*), and 17p11.2 microdeletion/duplication (*RAI1*). Notably, individuals carrying these P/LP CNVs exhibit highly variable clinical phenotypes. We also identified several P/LP CNVs that are less frequently reported in the literature, such as 15q11.2 deletion (BP1-BP2 region), 16p11.2 microdeletion/duplication (*HIRIP3*), 1p36.33 microdeletion (1p36 deletion syndrome region), 1q21.1 microdeletion/duplication (*GJA5*, *PRKAB2*, *CHD1L*), 8q21.11 microdeletion (*HEY1*), and 15q24 microdeletion (*ARID3B*). We acknowledge that 15q11.2 BP1-BP2 deletions exhibit incomplete penetrance and variable expressivity, and their clinical significance remains debated. Our classification as P/LP was based on the current ACMG guidelines and ClinGen dosage sensitivity recommendations. However, we recognize that some laboratories may conservatively classify these deletions as VOUS, and the implications for prenatal counseling should be approached with appropriate caution. All P/LP CNVs, including the 33 CNVs without a clear association with CHD, were reported to parents through post-test genetic counseling in accordance with institutional protocol.

A one-case discrepancy in the numerical counts of numerical abnormalities exists between Table 1 (karyotyping: 82 cases) and Table 2 (CMA: 81 cases). This reflects a classification difference: one case with a mosaic sex chromosome numerical abnormality, as determined by karyotyping, was further characterized by CMA as a complex Y chromosome structural rearrangement (with concurrent duplication and deletion), which is properly classified as a pathogenic CNV rather than a numerical abnormality. Consequently, this case was counted as a numerical abnormality in Table 1 but classified under P/LP CNVs in Table 2. This distinction highlights the complementary nature of the two technologies and does not affect the study’s main conclusions.

Balanced structural rearrangements were identified in 25 cases (4 translocations and 21 inversions), with 18 live births and 6 terminations. Their clinical significance in relation to fetal CHD remains uncertain, as they may be incidental findings or, less commonly, disrupt genes critical to cardiac development. From a genetic counseling perspective, parental karyotyping is essential to distinguish inherited from *de novo* rearrangements, as this directly informs recurrence risk. Notably, these 25 cases (2.0% of our cohort) would not have been detected by CMA alone, supporting the continued supplementary role of karyotyping in selected prenatal settings where balanced rearrangement detection is clinically relevant for recurrence risk counseling.

In this study, WES was performed on 131 CHD fetuses with negative karyotyping and CMA results. A total of 18 variants were identified, including 5 P, 11 LP, and 2 VOUS. Identified variants in genes, such as *Tab2* and *NONO,* were consistent with their associated CHD phenotypes. However, certain pathogenic variants (e.g., *PKP2* and *COL3A1*) exhibited phenotypic discordance, which may be attributed to expansion of the known clinical spectrum or the fact that some syndrome-associated features are not yet manifested in the fetal period. Notably, one fetus carried an NIPBL variant and presented with phenotypes highly overlapping with Cornelia de Lange syndrome (TOF with multiple severe anomalies). The NIPBL variant was classified as LP based on ACMG/ClinGen guidelines (16). The variant is a non-sense mutation satisfying PVS1. The fetal phenotype—TOF with multiple anomalies including vertebral and limb defects—is highly suggestive of Cornelia de Lange syndrome, supporting PP4 (30). Although parental samples were unavailable to confirm *de novo* status (PS2), the combination of PVS1 and PP4 is sufficient for an LP classification in the prenatal setting. We acknowledge that confirming *de novo* status would upgrade this variant to pathogenic, as reported in similar cases (31). When the known syndrome phenotype associated with a VOUS closely aligns with fetal ultrasound findings, careful consideration during multidisciplinary consultation is warranted. The higher WES diagnostic yield in our cohort (12.2%) compared to the 3–5% reported in unselected CHD cohorts is primarily attributable to our study setting. As a provincial-level tertiary referral center, our hospital receives complex cases referred from across the region, resulting in enrichment for severe and non-isolated CHD phenotypes with higher genetic risk.

Follow-up data showed that fetuses with pathogenic genetic abnormalities had a significantly higher rate of adverse pregnancy outcomes (90.7%) than those without (12.5%). Among the 16 live-born cases, 4 exhibited language or developmental delay and 1 had intellectual disability, including a child with a pathogenic *NONO* variant, whose phenotype aligned with the reported spectrum of intellectual disability (32, 33). These findings are consistent with studies linking CNVs to developmental delay (34, 35). Notably, the five live-born cases with 15q11.2 BP1-BP2 deletions all achieved favorable short-term outcomes without documented neurodevelopmental delays; however, given the well-documented incomplete penetrance and variable expressivity of this CNV, long-term follow-up remains warranted. In contrast, most fetuses with VOUS were live-born and showed no overt abnormalities during the initial postnatal period of 3–6 months, which aligns with previous observations (35). However, given that many neurodevelopmental and cardiac phenotypes may manifest later in childhood, this short follow-up window is insufficient to conclude that these VOUS are definitively benign. Continued long-term surveillance is warranted for these infants.

This study has several limitations. First, as a single-center retrospective study, selection bias and limited sample sizes for rare CHD types are unavoidable. Second, WES was performed only on probands, without parental confirmation. This limitation may have led to variant misclassification, as *de novo* status could not be confirmed for autosomal dominant conditions (e.g., the NIPBL variant was classified as LP based on PVS1 + PP4; confirmation of *de novo* status would have upgraded it to pathogenic), nor could we distinguish true compound heterozygosity from two variants in cis. Therefore, the designation “compound heterozygous” is used descriptively based on the fetal genotype alone, without implying confirmed phase or inheritance. Third, the relatively short postnatal follow-up period limited the assessment of long-term neurodevelopmental and cardiovascular outcomes. Fourth, given the retrospective design and the use of proband-only WES, our findings should be considered primarily hypothesis-generating. The observed genotype–phenotype associations require further validation before being translated into direct clinical decision-making algorithms. Fifth, all genomic data were aligned to GRCh37/hg19, the prevailing clinical standard during the study period. The CytoScan 750 K array annotations and our interpretation pipeline were designed for this assembly; therefore, its use is unlikely to have materially affected variant classification or CNV interpretation. Future studies should adopt the latest reference genome to ensure optimal accuracy. Sixth, WES was performed in only 10.9% of eligible cases due to logistical constraints, potentially introducing selection bias. Prospective studies with consecutive enrollment are needed to validate our findings.

## Conclusion

Fetal CHD is strongly associated with genetic abnormalities, which can be effectively detected via stepwise testing (karyotyping, CMA, WES) and are associated with adverse outcomes. Ultrasound reveals that genetic risk varies by CHD type, complexity, and extracardiac anomalies. Integrating detailed phenotypic information may inform more precise prenatal genetic risk stratification, though prospective validation is needed to establish clinical utility.

## Data Availability

The raw data supporting the conclusions of this manuscript will be made available by the authors, without undue reservation, to any qualified researcher.
