# Supplementary material for: Prenatal genetic findings and pregnancy outcomes in fetuses with congenital heart disease: a decade-long retrospective analysis at a tertiary hospital
Source: Front Med (Lausanne). 2026 Aug 17;13:1870270. doi: 10.3389/fmed.2026.1870270 (PMC13526937; doi:10.3389/fmed.2026.1870270)
Supplement: Supplementary file 1 [file Table_1.doc]

Supplementary **Table 1. 42 cases of P/LP CNVs with an established or likely causal relationship with CHD detected by CMA**

| No. | Ultrasound phenotype | Karyotype result | SNP array (CNV size) | Possible associated gene | Pregnancy outcome |
| --- | --- | --- | --- | --- | --- |
| 1 | VSD, fetal cleft palate, thymic hypoplasia | Normal | arr[GRCh37]22q11.21(18916843-21464764)x1 (2.5Mb) | 22q11.21 microdeletion | TOP |
| 2 | VSD, fetal NT thickening, cervical cystic hygroma | Normal | arr[GRCh37]22q11.21q11.23(19773308-23650420)x2-3 (3.8Mb) | 22q11.21q11.23 microduplication | TOP |
| 3 | VSD | Normal | arr[GRCh37]22q11.21(18648856-21461017)x3  (2.8Mb) | 22q11.21 microduplication | LB |
| 4 | VSD, thymic hypoplasia | Normal | arr[GRCh37]22q11.21(18916843-21464764)x1 (2.5Mb) | 22q11.21 microdeletion | TOP |
| 5 | VSD | Normal | arr[hg19]22q11.21(18,648,855-21,800,471)x1 (3.1Mb) | 22q11.21 microdeletion | TOP |
| 6 | TOF, thymic hypoplasia; bilateral clubfoot | Normal | arr[GRCh37]22q11.21(18916843-21800471)x1 (2.81Mb) | 22q11.21 microdeletion | TOP |
| 7 | TOF | Normal | arr[GRCh37]22q11.21(18631365-21800471)x1 (3.16Mb) | 22q11.21 microdeletion | TOP |
| 8 | TOF, small thymus | Normal | arr[GRCh37]22q11.21(18631365-21800471)x1 (3.2Mb) | 22q11.21 microdeletion | TOP |
| 9 | Truncus arteriosus | Normal | arr[GRCh37]22q11.21(18631364-20312661)x1 (1.68Mb) | 22q11.21 microdeletion | TOP |
| 10 | Pulmonary atresia, thymic hypoplasia, suspicion of DiGeorge syndrome | Normal | arr[GRCh37]22q11.21(18648856-21800471)x1 (3.1Mb) | 22q11.21 microdeletion | TOP |
| 11 | Pulmonary atresia | Normal | arr[hg19]22q11.21(18,919,477-21,800,471)x1 (2.8MB) | 22q11.21 microdeletion | TOP |
| 12 | Absent pulmonary valve syndrome | Normal | arr[hg19] 22q11.21(18,648,855-21,800,471)x1 (3.1Mb) | 22q11.21 microdeletion | TOP |
| 13 | HLHS, fetal NT thickening, cervical cystic hygroma | 44,X,der(6)t(6;22)(q26;q11.1),-22 | arr[GRCh37](X,22)x1,6q26q27(162434354-170914297)x1 (8.48Mb)  ,22q11.1q11.21(16888900-20312661)x1 (3.42Mb) | 22q11.1q11.21 microdeletion; 6q26q27 microdeletion | TOP |
| 14 | Interrupted aortic arch (type B), VSD | Normal | arr[hg19]22q11.21(18,631,364-21,800,471)x1 (3.1Mb) | 22q11.21 microdeletion | LB |
| 15 | Coarctation of the aorta | Normal | arr[GRCh37]22q11.21(18631364-21800471)x1 (3.1Mb) | 22q11.21 microdeletion | TOP |
| 16 | Right aortic arch | Normal | arr[GRCh37]22q11.21(18636749-21800471)x1 (3.1Mb) | 22q11.21 microdeletion | TOP |
| 17 | Aberrant right subclavian artery | Normal | arr[GRCh37]22q11.21(18648856-21915207)x3 (3.3Mb) | 22q11.21 microduplication | LB |
| 18 | Aberrant right subclavian artery | Normal | arr[GRCh37]22q11.21(18648856-21800471)x1 (3.15Mb) | 22q11.21 microdeletion | TOP |
| 19 | Aberrant right subclavian artery | Normal | arr[hg19]22q11.21(18,631,364-21,800,471)x1 (3.2Mb) | 22q11.21 microdeletion | TOP |
| 20 | Aberrant right subclavian artery, bilateral renal collecting system dilation | Normal | arr[hg19]22q11.21(18,916,842- 21,800,471)x1 (2.9Mb) | 22q11.21 microdeletion | LB |
| 21 | PLSVC | Normal | arr[hg19]22q11.21(18,919,477-21,800,471)x1 (2.9Mb) | 22q11.21 microdeletion | TOP |
| 22 | VSD | Normal | arr[hg19]17p11.2(16,727,490-20,433,723)x1 (3.7Mb) | 17p11.2 microdeletion | TOP |
| 23 | Right aortic arch | Normal | arr[hg19]17p11.2(16,615,982-18,922,171)x3 dn  (2.3Mb) | 17p11.2 microduplication | LB |
| 24 | VSD | Normal | arr[hg19]7q11.21q11.23(66,419,483-74,312,482)x1 (7.89Mb) | 7q11.21q11.23 microdeletion | TOP |
| 25 | Truncus arteriosus | 46,XN,del(5)(p14) | arr[hg19]5p15.33p14.3(113,576-22,955,916)x1 (22.8Mb) | 5p15.33p14.3 microdeletion | TOP |
| 26 | Aortic stenosis | 46,XN,del(4)(p14) | arr[hg19]4p16.3p14(68,345-37,141,015)x1 (37Mb) | 4p16.3p14 microdeletion | TOP |
| 27 | VSD | 46,XN,del(11)(q23.3) | arr[hg19]11q23.3q25(120,583,764-134,937,416)x1 (14.3Mb) | 11q23.3q25 microdeletion | TOP |
| 28 | VSD, mild tricuspid regurgitation | Normal | arr[GRCh37]15q11.2(22770422-23290788)x1 (520Kb) | 15q11.2 microdeletion | LB |
| 29 | VSD | Normal | arr[GRCh37]15q11.2(22770422-23082237)x1 (312Kb) | 15q11.2 microdeletion | LB |
| 30 | Aberrant right subclavian artery | Normal | arr[GRCh37]15q11.2(22770422-23206905)x1 (518Kb) | 15q11.2 microdeletion | LB |
| 31 | Aberrant right subclavian artery | Normal | arr[GRCh37]15q11.2q13.1(22770422-28928730)x1 (6.16Mb) | 15q11.2q13.1 microdeletion | TOP |
| 32 | Mirror-image dextrocardia | Normal | arr[GRCh37]15q11.2(22770422-23082237)x1  (312Kb) | 15q11.2 microdeletion | LB |
| 33 | VSD, moderate tricuspid regurgitation | Normal | arr[GRCh37]15q11.2(22770421-23277436)x1 (507Kb) | 15q11.2 microdeletion | LB |
| 34 | TOF | Normal | arr[GRCh37]16p11.2(28384464-29388495)x1 (1Mb) | 16p11.2 microdeletion | TOP |
| 35 | TOF | Normal | arr[hg19]16p11.2(29,428,531-30,190,029)x1 dn (623Kb) | 16p11.2 microdeletion | TOP |
| 36 | TOF | Normal | arr[hg19]16p11.2(28,786,703-29,032,280)x3   (246Kb) | 16p11.2 microduplication | TOP |
| 37 | Aberrant right subclavian artery | Normal | arr[GRCh37]16p11.2(29591327-30190029)x3 (599Kb) | 16p11.2 microduplication | TOP |
| 38 | VSD | Normal | arr[GRCh37]1p36.33p36.32(849466-3101616)x1 (2.2Mb) | 1p36.33p36.32 microdeletion | TOP |
| 39 | VSD, fetal ductus venosus-systemic venous shunt | 46,XN,del(1)(p36.3) | arr [hg19]1p36.33p36.23(849,466-8.056.475)x1 (7.2Mb) | 1p36.33p36.23 microdeletion | TOP |
| 40 | Aberrant right subclavian artery | Normal | arr[GRCh37]1q21.1q21.2(146023923-147832190)x1 (1.8Mb) | 1q21.1q21.2 microdeletion | TOP |
| 41 | VSD, cleft palate, nasal bone hypoplasia | 46,XN,del(8)(q21.1q22.2) | arr[hg19]8q21.11q22.1(76,860,026-95,799,552)x1 dn (18.9Mb) | 8q21.11q22.1 microdeletion | TOP |
| 42 | VSD | Normal | arr[hg19]15q24.1q24.2(72,965,465-75,567,135)x1 (2.6Mb) | 15q24.1q24.2 microdeletion | TOP |

Abbreviations: VSD, ventricular septal defect; TOF, tetralogy of Fallot; HLHS, hypoplastic left heart syndrome; NT, nuchal translucency; CMA, chromosomal microarray analysis; P/LP CNVs, pathogenic/likely pathogenic copy number variations; PLSVC, persistent left superior vena cava; LB, live birth; TOP, termination of pregnancy.

Supplementary Table 2. 27 cases of VOUS CNVs detected by CMA

| No. | Ultrasound phenotype | Karyotype result | SNP array (CNV size) | Pregnancy outcome | |
| --- | --- | --- | --- | --- | --- |
| 1 | VSD, nasal bone hypoplasia, small thymus | 46,X?,t(2;3)(q37;p13)dn | arr[GRCh37]3p14.2(61233908-62675592)x1 (1.4Mb) | TOP | |
| 2 | VSD, nasal bone hypoplasia, right hand polydactyly | Normal | arr[GRCh37]16p13.11(14893699-16495076)x3 (1.6Mb) | | LB |
| 3 | VSD | Normal | arr[GRCh37]3q24(143561987-146068738)x1 (2.5Mb) | | TOP |
| 4 | VSD | Normal | arr[GRCh37]2p16.1(56788428-58572494)x4 (1.78Mb) | | LB |
| 5 | VSD | Normal | arr[GRCh37]11q23.3(116161830-116677848)x1 (516Kb) | | LB |
| 6 | VSD | Normal | arr[GRCh37]1q21.1(144887880-146011861)x1 (1.1Mb) | | TOP |
| 7 | VSD | Normal | arr[GRCh37]3p14.3p14.2(57885087-61819903)x1- (3.9Mb) | | LB |
| 8 | VSD, fetal cleft lip and palate | Normal | arr[GRCh37]4q35.1q35.2(186031030-189191908)x3 (3.1Mb) | | LB |
| 9 | VSD, nasal bone hypoplasia | Normal | arr[GRCh37]22q11.21(21058887-21464764)x1 (406kb) | | LB |
| 10 | VSD | Normal | arr[hg19]13q14.2(48,515,697-50,122,839)x3 (1.6Mb) | | LB |
| 11 | VSD | Normal | arr[hg19]1q24.2(170,154,213-170,678,832)x3 (525Kb) | | LB |
| 12 | VSD | Normal | arr[hg19]9q21.33q22.1(89,868,507-90,975.015)x3 (1.1 Mb ) | | LB |
| 13 | VSD | Normal | arr[hg19]13q14.11(43,115,979-43,733,172)x3 (617Kb) | | LB |
| 14 | PLSVC | Normal | arr[GRCh37]22q13.31q13.33(47978042-50194178)x3 (2.2Mb) | | LB |
| 15 | PLSVC, possible right pelvic ectopic kidney | Normal | arr[hg19]10q21.1(57,335,763-59,029.364)x1 (1.69Mb) | | LB |
| 16 | PLSVC | Normal | arr[hg19]15q11.2(22,770,421-23,276,833)x1 (506Kb) | | LB |
| 17 | PLSVC | Normal | arr[hg19]2p25.3p11.2(50,813-87,053,152)hmz arr[hg19]2q11.1q37.3(95,550,957-242,773,583)hmz (234.2Mb) | | LB |
| 18 | Pulmonary stenosis | Normal | arr[GRCh37]6p21.1p12.1(43221909-55129381)x2 hmz 1 (11.9Mb) | | TOP |
| 19 | Pulmonary stenosis | Normal | arr[GRCh37]4p13p12(41623048-45338240)x1 (3.7Mb) | | TOP |
| 20 | Pulmonary atresia, nasal bone hypoplasia, thymus not visualized | Normal | arr[GRCh37]Xp22.33p11.1(169922-58227320)x2 hmz Xq11.1q28(62036671-155233846)x2 hmz (151.25Mb) | | TOP |
| 21 | Aberrant right subclavian artery | Normal | arr[GRCh37]2q11.1q11.2(95327874-97881361)x3 (2.5Mb) | | TOP |
| 22 | Aberrant right subclavian artery | Normal | arr[GRCh37]5q13.3(7512616-756755)x1  (572Kb) | | LB |
| 23 | TOF | Normal | arr[hg19]16p12.3(19,333,917-20,487,932)x1 (1.1Mb) | | TOP |
| 24 | Coarctation of the aorta, anomalous ductus venosus connection | 46,X?,inv(9)(p12q13) | arr[GRCh37]16q22.2q23.2(71463698-79614082)x3 (8.15Mb) | | LB |
| 25 | Aortic stenosis | Normal | arr[hg19]22q11.21(20,310,410-21,464.764)x3 (1.15Mb) | | LB |
| 26 | Tricuspid stenosis | Normal | arr [hg19]10q21.1(59,095,330-60,684,488)x1 (1.5Mb) | | LB |
| 27 | Anomalous pulmonary venous connection | Normal | arr[hg19]10q11.22q11.23(46,975,076-51,795,569)x3 (4.8Mb) | | TOP |

Abbreviations: VSD, ventricular septal defect; TOF, tetralogy of Fallot; VOUS, variants of uncertain significance; CMA, chromosomal microarray analysis; CNV, copy number variation; PLSVC, persistent left superior vena cava; LB, live birth; TOP, termination of pregnancy.

Supplementary Table 3. Gene variants detected by WES

| No. | Ultrasound phenotype | Gene | Mutation site and amino acid change | Exon/Intron | Chromosomal position | Zygosity | Classification |
| --- | --- | --- | --- | --- | --- | --- | --- |
| 1 | VSD, myocardial thickening, mild tricuspid regurgitation | *NONO* (NM-001145408.1) | c.457C>T p.Arg153* | exon 6 | chrX-70514185-C-T | Hemizygous | P=PVS1+PM2-Supporting+PS2 |
| 2 | Pulmonary stenosis, NT thickening, left ventricular hyperechogenic focus, bilateral renal collecting system separation | *MYLK* (NM-053025.3) | c.589-2A>G | intron 7 | chr3-123456392-T-C | Heterozygous | P=PVS1+PM2-Supporting+PS2 |
| 3 | Pulmonary stenosis, VSD | *ZEB2* (NM-014795.4) | c.2083C>T p.Arg695* | exon 8 | chr2-145156671-G-A | Heterozygous | P=PVS1+PM2-Supporting+PS4+PS2 |
| 4 | Right aortic arch, fetal scalp edema, right hydronephrosis with ureteral dilation, gallbladder not visualized | *KMT2D* (NM-003482.3) | c.15568C>T p.Gln5190* | exon 49 | chr12-49420181-G-A | Heterozygous | P=PVS1+PM2-Supporting+PM3 |
| 5 | Tortuous course of aortic arch branches, ductus arteriosus, and descending aorta; slender long bones of extremities; slender fingers and toes; inguinal hernia | *EFEMP2* (NM-016938.4) | c.499G>T  p.Glu167* | exon6 | chr11-65637700-C-A | Heterozygous | LP=PVS1+PM2-Supporting |
| 6 | Coarctation of the aorta, severe tricuspid regurgitation | *TAB2* (NM-015093.4) | c.694delC p.His232Ilefs*11 | exon 5 | chr6-149699745 del | Heterozygous | LP=PVS1+PM2-Supporting |
| 7 | HLHS, fetal hydrops, cervical cystic hygroma, pleural effusion, ascites | *DSP* (NM-004415.2) | c.3384-3388delGGAAG p.Glu1129Glnfs*3 | exon 23 | chr6-7579807-7579811 del | Heterozygous | LP=PVS1+PM2-Supporting |
| 8 | Rhabdomyoma, bilateral clubfoot | *TSC2* (NM-000548.5) | c.1864C>T p.Arg622Trp | exon18 | chr16-2121535-C-T | Heterozygous | LP=PVS1+PM2-Supporting |
| 9 | Complete TGA | *PKP2* (NM-004572.3) | c.1170+1G>A | intron 4 | chr12-33021860-C-T | Heterozygous | P=PVS1+PM2-Supporting+PS4 |
| 10 | Complete TGA, VSD | *KDR* (NM-002253.2) | c.2615-2A>G | Intron 18 | chr4-55962511-T-C | Heterozygous | LP=PVS1+PM2-Supporting |
| 11 | TOF | TTC37 (NM-014639.3) | c.3426-3427insA p.Ala1143Serfs*4 | exon 33 | chr5-94834211 dup | Heterozygous | LP=PVS1+PM2-Supporting |
| 12 | DORV | *NODAL* (NM-018055.4) | c.670-671insGACTCGAAAATCTTACCAACTCATAGAGTTGGGGTGAGAATTCGA p.Glu224-Leu347delinsGlyLeuGluAsnLeuThrAsnSer | exon 2 | chr10-72195263 | Heterozygous | LP=PVS1+PM2-Supporting |
| 13 | Pulmonary atresia, fetal hydrocephalus, cleft lip and palate, slightly enhanced echogenicity of gastric wall and local intestinal wall, narrow right umbilical artery | TCAP (NM-003673.3) | c.460-472delCGCTCCCTGTCCC p.Arg154Alafs*30 | exon 2 | chr17-37822318-37822330 del | Heterozygous | LP=PVS1+PM2-Supporting |
| 14 | Pulmonary atresia, coronary artery fistula | *COL3A1* (NM-000090.4) | c.5del p.Met2ArgfsTer64 c.7-8insC p.Ser3Thrfs*27 | exon 1 exon 1 | chr2-189839220 del chr2-189839222-189839223 ins C | Heterozygous | LP=PVS1+PM2-Supporting |
| 15 | Anomalous pulmonary venous connection | NEK1 (NM-012224.4) | c.1908delA p.Val637Cysfs*34 | exon 21 | chr4-170428203 del | Heterozygous | LP=PVS1+PM2-Supporting |
| 16 | TOF | *TRPM4* (NM-017636.3) | c.3504-3514delCGAACAGCGCC p.Gln1170Serfs*10 | exon 23 | chr19-49714314 | Heterozygous | VOUS=PVS1-Moderate+PM2-Supporting |
| 17 | Complete TGA, fetal nasal bone hypoplasia | SON (NM-032195.1) | c.6754A>T p.Lys2252* | exon 7 | chr21-34932279-A-T | Heterozygous | VOUS=PM4+PM2-Supporting |
| 18 | TOF, vertebral anomaly; bilateral renal agenesis; right radial and ulnar anomalies, abnormal hand posture; bilateral tibial and fibular anomalies, feet not visualized | *NIPBL* (NM-133433.4) | c.1319del p.Asn440Thrfs*41 c.1302-1303insT p.Pro435Serfs*33 c.1325C>G p.Ser442* | exon 9 exon 9 exon 9 | chr5-36976328 del chr5-36976311-36976312 ins T chr5-36976334-C-G | Heterozygous | LP=PVS1+PP4 |

Supplementary Table 4 Distribution of 1354 cases of CHD

| Category | Number (%) | Type | Number (%) | LB | TOP | Lost to follow-up |
| --- | --- | --- | --- | --- | --- | --- |
| I. Septal defects | 567(41.9) | VSD | 555(41.0) | 466 | 84 | 5 |
|  |  | AVSD | 11(0.8) | 2 | 9 | 0 |
|  |  | Single ventricle | 1(0.1) | 0 | 1 | 0 |
| II. Conotruncal anomalies | 92(6.8) | TOF | 67(5.0) | 26 | 40 | 1 |
|  |  | DORV | 11(0.8) | 1 | 10 | 0 |
|  |  | TGA | 11(0.8) | 4 | 7 | 0 |
|  |  | Truncus arteriosus | 3(0.2) | 0 | 3 | 0 |
| III. Right heart anomalies | 49(3.6) | Pulmonary stenosis | 22(1.6) | 12 | 8 | 2 |
|  |  | Pulmonary atresia | 13(1.0) | 1 | 12 | 0 |
|  |  | Ebstein anomaly | 4(0.3) | 1 | 3 | 0 |
|  |  | Tricuspid stenosis | 3(0.2) | 2 | 1 | 0 |
|  |  | Right heart hypoplasia | 2(0.2) | 0 | 2 | 0 |
|  |  | Anomalous origin of pulmonary artery | 2(0.2) | 1 | 1 | 0 |
|  |  | Tricuspid atresia | 1(0.1) | 0 | 1 | 0 |
|  |  | Pulmonary artery sling | 1(0.1) | 1 | 0 | 0 |
|  |  | Absent pulmonary valve syndrome | 1(0.1) | 0 | 1 | 0 |
| IV. Left heart anomalies | 89(6.6) | Coarctation of the aorta | 67(5.0) | 42 | 22 | 3 |
|  |  | Aortic stenosis | 7(0.5) | 3 | 4 | 0 |
|  |  | Interrupted aortic arch | 5(0.4) | 3 | 2 | 0 |
|  |  | Anomalies of aortic arch and branches | 5(0.4) | 4 | 1 | 0 |
|  |  | HLHS | 4(0.3) | 0 | 4 | 0 |
|  |  | Mitral stenosis | 1(0.1) | 0 | 1 | 0 |
| V. Venous return anomalies | 123(9.1) | PLSVC | 118(8.7) | 103 | 15 | 0 |
|  |  | Anomalous pulmonary venous connection | 5(0.4) | 1 | 4 | 0 |
| VI. Cardiac position anomalies | 21(1.6) | Dextrocardia | 11(0.8) | 8 | 3 | 0 |
|  |  | Heterotaxy syndrome | 10(0.7) | 4 | 5 | 1 |
| VII. Other cardiac anomalies | 413(30.5) | Vascular ring | 395(29.2) | 347 | 46 | 2 |
|  |  | Absent aortic/pulmonary valve | 6(0.4) | 0 | 6 | 0 |
|  |  | Coronary artery fistula | 4(0.3) | 2 | 1 | 1 |
|  |  | Ventricular diverticulum | 2(0.2) | 1 | 0 | 1 |
|  |  | Noncompaction cardiomyopathy | 2(0.2) | 1 | 1 | 0 |
|  |  | Rhabdomyoma | 2(0.2) | 0 | 2 | 0 |
|  |  | Endocardial fibroelastosis | 1(0.1) | 0 | 1 | 0 |
|  |  | Criss-cross heart | 1(0.1) | 0 | 1 | 0 |
| Total |  |  | 1354 | 1036 | 302 | 16 |

Abbreviation: TOP: termination of pregnancy; VSD: ventricular septal defect; AVSD: atrioventricular septal defect; PLSVC: persistent left superior vena cava;TOF: tetralogy of Fallot; DORV: double outlet right ventricle; TGA: transposition of the great arteries; HLHS: hypoplastic left heart syndrome.

Supplementary Table 5. Detection of pathogenic genetic abnormalities in different types of CHD

| CHD type | Number (%) | Pathogenic genetic abnormalities | | |
| --- | --- | --- | --- | --- |
|  |  | Total abnormal (%) | Numerical chromosomal abnormalities (%) | P/ LP CNVs |
|  |  |  |  | (%) |
| PLSVC | 118(8.7) | 5(4.2) | 2 (1.7) | 3(2.5) |
| Transposition of the great arteries | 11(0.8) | 0(0) | 0 (0) | 0(0) |
| Dextrocardia | 11(0.8) | 1(9.1) | 0 (0) | 1(9.1) |
| Heterotaxy syndrome | 10(0.7) | 0(0) | 0 (0) | 0(0) |
| Vascular ring | 395(29.2) | 40(10.1) | 19 (4.8) | 21(5.3) |
| VSD | 555(41.0) | 50(9.0) | 24 (4.3) | 26(4.7) |
| Pulmonary stenosis | 22(1.6) | 1(4.6) | 0 (0) | 1(4.6) |
| Coarctation of the aorta | 67(5.0) | 9(13.4) | 5 (7.5) | 4(6.0) |
| Coronary artery fistula | 4(0.3) | 1(25.0) | 1 (25.0) | 0(0) |
| Ebstein anomaly | 4(0.3) | 1(25.0) | 1 (25.0) | 0(0) |
| DORV | 11(0.8) | 3(27.3) | 3 (27.3) | 0(0) |
| TOF | 67(5.0) | 17(25.4) | 10 (14.9) | 7(10.5) |
| Anomalous pulmonary venous connection | 5(0.4) | 1(20.0) | 0 (0) | 1(20.0) |
| Anomalies of aortic arch and branches | 5(0.4) | 1(20.0) | 0 (0) | 1(20.0) |
| Interrupted aortic arch | 5(0.4) | 2(40.0) | 1 (20.0) | 1(20.0) |
| AVSD | 11(0.8) | 5(45.5) | 5 (45.5) | 0(0) |
| Pulmonary atresia | 13(1.0) | 4(30.8) | 1 (7.7) | 3(23.1) |
| Aortic stenosis | 7(0.5) | 3(42.9) | 2 (28.6) | 1(14.3) |
| Absent aortic/pulmonary valve | 6(0.4) | 4(66.7) | 4 (66.7) | 0(0) |
| Tricuspid stenosis | 3(0.2) | 1(33.3) | 1 (33.3) | 0(0) |
| HLHS | 4(0.3) | 3(75.0) | 2 (50.0) | 1(25.0) |
| Truncus arteriosus | 3(0.2) | 3(100) | 0 (0) | 3(100) |
| Absent pulmonary valve syndrome | 1(0.07) | 1(100) | 0 (0) | 1(100) |
| Anomalous origin of pulmonary artery | 2(0.15) | 0(0) | 0 (0) | 0(0) |
| Rhabdomyoma | 2(0.2) | 0(0) | 0 (0) | 0(0) |
| Noncompaction cardiomyopathy | 2(0.2) | 0(0) | 0 (0) | 0(0) |
| Ventricular diverticulum | 2(0.2) | 0(0) | 0 (0) | 0(0) |
| Right heart hypoplasia | 2(0.2) | 0(0) | 0 (0) | 0(0) |
| Single ventricle | 1(0.1) | 0(0) | 0 (0) | 0(0) |
| Mitral stenosis | 1(0.1) | 0(0) | 0 (0) | 0(0) |
| Pulmonary artery sling | 1(0.1) | 0(0) | 0 (0) | 0(0) |
| Tricuspid atresia | 1(0.1) | 0(0) | 0 (0) | 0(0) |
| Criss-cross heart | 1(0.1) | 0(0) | 0 (0) | 0(0) |
| Endocardial fibroelastosis | 1(0.1) | 0(0) | 0 (0) | 0(0) |
| Total | 1354 | 156 | 81 | 75 |
|  | | | | |

Abbreviations: CHD, congenital heart disease; P/LP CNVs, pathogenic/likely pathogenic copy number variations; PLSVC: persistent left superior vena cava; VSD, ventricular septal defect; TOF, tetralogy of Fallot; TGA, transposition of the great arteries; DORV, double outlet right ventricle; HLHS, hypoplastic left heart syndrome; AVSD, atrioventricular septal defect.

Supplementary Table 6 Genetic variants significantly associated with CHD ultrasound phenotypes: Clinical phenotypes and references

| No. | Ultrasound phenotype | WES result | Clinical phenotype of variant gene | Concordance between ultrasound and clinical phenotype | References |
| --- | --- | --- | --- | --- | --- |
| 1 | VSD, tricuspid regurgitation | *NONO* gene mutation in exon 6, c.457C>T, p.Arg153* | *NONO* gene mutations are associated with X-linked syndrome 34, intellectual developmental disorder, characterized by psychomotor developmental delay, intellectual disability with poor speech, facial dysmorphism, thickened corpus callosum, atrial and ventricular septal defects | Concordant; gene mutation classified as pathogenic | doi:10.1136/jmedgenet-2016-104039.  .doi:10.1002/ajmg.a.61466. |
| 2 | Pulmonary stenosis, VSD | *MYLK* gene mutation in intron 7, c.589-2A>G | *MYLK* gene mutations are associated with autosomal dominant familial thoracic aortic aneurysm or dissection, mainly characterized by extensive arterial dissection involving many arteries including renal, iliac, superior mesenteric, subclavian, and innominate arteries | Concordant; classified as pathogenic | doi:10.1186/s13023-018-0769-7. |
| 3 | Pulmonary stenosis, VSD | *ZEB2* gene mutation in exon 8, c.2083C>T, p.Arg695* | *ZEB2* gene mutations are associated with autosomal dominant Mowat-Wilson syndrome, with CHD manifestations including atrial or ventricular septal defect, pulmonary stenosis, coarctation of the aorta, TOF, aortic valve abnormalities, and pulmonary artery sling with or without tracheal stenosis | Concordant; classified as pathogenic | doi:10.3390/ijms25052838 |
| 4 | Right aortic arch, aberrant left subclavian artery, scalp edema | *KMT2D* gene mutation in exon 49, c.15568C>T, p.Gln5190* | *KMT2D* gene mutations cause autosomal dominant Kabuki syndrome 1, mainly characterized by lower eyelid eversion, depressed nasal tip, prominent ears, characteristic facial dysmorphism, skeletal and branchial arch abnormalities, as well as single atrium, single ventricle, VSD, TOF, coarctation of the aorta | Concordant; classified as pathogenic | doi:10.3390/diagnostics14080846. |
| 5 | Tortuous course of aortic arch branches, ductus arteriosus, and descending aorta; inguinal hernia; slender long bones of extremities; slender fingers and toes | *EFEMP2* gene mutation in exon 6, c.499G>T, p.Glu167* | *EFEMP2* gene mutations cause autosomal recessive cutis laxa type IB, mainly characterized by cutis laxa, arterial aneurysms, arterial tortuosity, joint laxity, birth fractures, and emphysema | Concordant; classified as pathogenic | doi:10.3389/fgene.2024.1453195 |
| 6 | Coarctation of the aorta, VSD, severe tricuspid regurgitation | *TAB2* gene mutation in exon 5, c.694delC, p.His232Ilefs*11 | *TAB2* gene mutations cause autosomal dominant non-syndromic CHD, mainly involving valves, also including septal defects, aneurysms, coarctation of the aorta, TOF | Concordant; classified as pathogenic | doi:10.1186/s12872-020-01404-5.  doi:10.1111/cge.13032. |
| 7 | HLHS, fetal hydrops | *DSP* gene mutation in exon 23, c.3384-3388delGGAAG, p.Glu1129Glnfs*3 | *DSP* gene mutations are associated with autosomal recessive dilated cardiomyopathy with woolly hair and keratoderma, mainly characterized by focal and diffuse palmoplantar keratoderma, hyperkeratotic plaques on trunk and limbs, woolly hair and variable alopecia, dilated left ventricular cardiomyopathy | Concordant; classified as pathogenic | doi:10.1186/s12881-020-0955-z.  doi:10.1111/ced.12226. |
| 8 | Rhabdomyoma, bilateral clubfoot | *TSC2* gene mutation in exon 18, c.1864C>T, p.Arg622Trp | *TSC2* gene mutations cause autosomal dominant tuberous sclerosis-2, characterized by hamartomas in multiple organ systems including brain, skin, heart, kidneys, and lungs | Concordant; classified as pathogenic | doi:10.1016/S0140-6736(08)61279-9 |
| 9 | Complete TGA | *PKP2* gene mutation in intron 4, c.1170+1G>A | *PKP2* gene mutations are associated with autosomal dominant arrhythmogenic right ventricular dysplasia, mainly characterized by congestive heart failure in infancy or early childhood, ventricular tachyarrhythmias, and sudden cardiac death | Discordant; classified as pathogenic | doi:10.1093/eurheartj/ehl380  doi:10.1161/01.cir.65.2.384 |
| 10 | Complete TGA, VSD | *KDR* gene mutation in intron 18, c.2615-2A>G | *KDR* gene mutations are associated with autosomal dominant infantile capillary hemangioma and tetralogy of Fallot, mainly characterized by vascular anomalies, ventricular septal defect, pulmonary stenosis, absent pulmonary valve syndrome, and double outlet right ventricle | Concordant; classified as pathogenic | doi:10.1038/s41436-021-01212-y.  doi:10.1007/s10528-024-10901-7. |
| 11 | TOF | *TTC37* gene mutation in exon 33, c.3426-3427insA, p.Ala1143Serfs*4 | TTC37 gene mutations are associated with autosomal recessive trichohepatoenteric syndrome, mainly characterized by infantile diarrhea, immunodeficiency, liver disease, trichorrhexis nodosa, facial dysmorphism, aortic regurgitation, peripheral pulmonary stenosis, ventricular septal defect, and tetralogy of Fallot | Largely concordant; classified as pathogenic | doi:10.1053/j.gastro.2010.02.010.  doi:10.21037/tp-21-574 |
| 12 | DORV, VSD | *NODAL* gene mutation in exon 2, c.670-671ins[68], p.Glu224-Leu347delins[13] | *NODAL* gene mutations cause autosomal dominant congenital heart disease, mainly characterized by pulmonary atresia, transposition of the great arteries, double outlet right ventricle, ventricular septal defect, single ventricle, anomalous pulmonary venous return, and atrial isomerism | Concordant; classified as pathogenic | doi:10.1093/hmg/ddn411 |
| 13 | Pulmonary atresia, VSD, cleft lip and palate, hydrocephalus | *TCAP* gene mutation in exon 2, c.460-472del, p.Arg154Alafs*30 | *TCAP* gene mutations are associated with autosomal dominant hypertrophic cardiomyopathy and autosomal recessive limb-girdle muscular dystrophy, mainly characterized by left ventricular hypertrophy, atrial enlargement with severe diastolic dysfunction, and limb muscle atrophy | Largely discordant; classified as pathogenic | doi:10.1093/ehjcr/ytaf180.  doi:10.1016/j.nmd.2017.05.017 |
| 14 | Pulmonary atresia, coronary artery fistula | *COL3A1* gene mutation in exon 1, c.5del, p.Met2ArgfsTer64 | *COL3A1* gene mutations are associated with autosomal recessive polymicrogyria and Ehlers-Danlos syndrome, mainly characterized by polymicrogyria, developmental delay, connective tissue defects, vascular fragility, aortic aneurysm, arterial rupture and dissection | Largely discordant; classified as pathogenic | doi:10.1002/mgg3.70095.  doi:10.1136/jmedgenet-2016-104421. |
| 15 | Total anomalous pulmonary venous connection | *NEK1* gene mutation in exon 21, c.1908delA, p.Val637Cysfs*34 | *NEK1* gene mutations are associated with autosomal recessive short-rib thoracic dysplasia with or without polydactyly, mainly characterized by thoracic constriction, short ribs, shortened tubular bones, cleft lip/palate, neurological abnormalities, mitral regurgitation, and tricuspid regurgitation | Discordant; classified as candidate variant of uncertain significance | doi:10.1002/ajmg.c.31336  doi:10.1038/jhg.2016.157 |
| 16 | TOF | *TRPM4* gene mutation in exon 23, c.3504-3514del, p.Gln1170Serfs*10 | *TRPM4* gene mutations are associated with autosomal dominant familial heart block type IB and Brugada syndrome, mainly characterized by sinus node dysfunction, atrioventricular or right bundle branch block, and ventricular tachyarrhythmias | Largely discordant; classified as pathogenic | doi:10.1080/00015385.2020.1790823.  doi:10.1002/humu.21599. |
| 17 | Complete TGA, nasal bone hypoplasia | *SON* gene mutation in exon 7, c.6754A>T, p.Lys2252* | *SON* gene mutations are associated with autosomal dominant ZTTK syndrome, mainly characterized by psychomotor developmental delay and intellectual disability, with characteristic facial dysmorphism, musculoskeletal and visual abnormalities, hearing loss, urogenital and cardiac defects | Concordant; classified as pathogenic | doi:10.1159/000546621. |
| 18 | TOF, vertebral and limb anomalies, bilateral renal agenesis | *NIPBL* gene mutation in exon 9, c.1319del p.Asn440Thrfs41; c.1302-1303insT p.Pro435Serfs33; c.1325C>G p.Ser442* | *NIPBL* gene mutations are associated with autosomal dominant Cornelia de Lange syndrome, mainly characterized by arched eyebrows, anteverted nares, prominent maxilla, long philtrum, thin lips, characteristic facial dysmorphism, upper limb anomalies, cardiac septal defects, growth retardation, and intellectual disability | Discordant; classified as likely pathogenic | doi:10.1172/JCI178355. |

Abbreviations: CHD, congenital heart disease; VSD, ventricular septal defect; TOF, tetralogy of Fallot; HLHS, hypoplastic left heart syndrome; TGA, transposition of the great arteries; DORV, double outlet right ventricle; WES, whole exome sequencing; p., protein; c., coding DNA; del, deletion; ins, insertion; fs, frameshift; *, stop codon.

Supplementary Table 7 Follow-up of 16 live-born fetuses with pathogenic genetic abnormalities.

| No. | Ultrasound phenotype | Genetic result | Pregnancy Outcome | Follow-up |
| --- | --- | --- | --- | --- |
| 3 a | VSD | CMA(pCNVs 22q11.21 microduplication) | Term live birth, Apgar score 10, birth weight 3890g | Generally in good condition |
| 14a | Interrupted aortic arch (type B), VSD | CMA(pCNVs 22q11.21 microdeletion) | Term live birth, Apgar score 10, birth weight 2800g | Live birth, died at 1 month of age |
| 17a | Aberrant right subclavian artery | CMA(pCNVs 22q11.21 microduplication) | Term live birth, Apgar score 10, birth weight 3170g | Generally in good condition |
| 20a | Aberrant right subclavian artery, bilateral renal collecting system dilation | CMA(pCNVs 22q11.21microdeletion) | Term live birth, Apgar score 10, birth weight 3490g | Speech delay |
| 28a | VSD, mild tricuspid regurgitation | CMA(pCNVs 15q11.2 microdeletion) | Term live birth, Apgar score 10, birth weight 2925g | Generally in good condition |
| 29a | VSD | CMA(pCNVs 15q11.2 microdeletion) | Term live birth, Apgar score 10, birth weight 3220g | Generally in good condition |
| 30a | Aberrant right subclavian artery, mild tricuspid regurgitation | CMA(pCNVs 15q11.2microdeletion) | Term live birth, Apgar score 10, birth weight 2690g | Generally in good condition |
| 32a | Mirror-image dextrocardia | CMA(pCNVs 15q11.2microdeletion) | Term live birth, Apgar score 10, birth weight 3390g | Generally in good condition |
| 33a | VSD, moderate tricuspid regurgitation | CMA(pCNVs 15q11.2microdeletion) | Term live birth, Apgar score 10, birth weight 2520g | Generally in good condition |
| 23a | Right aortic arch | CMA(lpCNVs 17p11.2 microduplication) | Preterm birth, Apgar score 10, birth weight 1980g | Intellectual disability |
| 1b | VSD, myocardial thickening, mild tricuspid regurgitation | WES(P | Term live birth, Apgar score 10, birth weight 2510g | Growth and developmental delay |
|  |  | NONO) |  |  |
| 11b | TOF | WES(LP | Term live birth, Apgar score 9, birth weight 2810g | Post-surgery, generally in good condition |
|  |  | TTC37) |  |  |
| - | Aberrant right subclavian artery | CMA(pCNVs 17p12 microduplication) | Term live birth, Apgar score 10, birth weight 3325g | Growth and developmental delay |
| - | Aberrant right subclavian artery | CMA(lpCNVs 1p36.21p35.2 microduplication) | Term live birth, Apgar score 10, birth weight 2625g | Generally in good condition |
| - | VSD, mild tricuspid regurgitation | CMA(pCNVs Xq28 microdeletion) | Term live birth, Apgar score 10, birth weight 3685g | Speech delay |
| - | Right aortic arch | CMA(lpCNVs 18q22.3q23 microduplication) | Term live birth, Apgar score 10, birth weight 2845g | Generally in good condition |

Note: a: Corresponds to the case number in Supplementary Table 1; b: Corresponds to the case number in Supplementary Table 3.

Supplementary Tables 8. Follow-up of 19 live-born fetuses with VOUS results by CMA and WES

| No. | Ultrasound phenotype | Genetic result | Pregnancy outcome | Follow-up |
| --- | --- | --- | --- | --- |
|  |  |  |  |  |
| 2* | VSD, nasal bone hypoplasia, right hand polydactyly | CMA(VOUS) | 34-week preterm birth, Apgar score 10, birth weight 1325g | Generally in good condition |
| 4* | VSD | CMA(VOUS) | Term live birth, Apgar score 10, birth weight 3370g | Generally in good condition |
| 5* | VSD | CMA(VOUS) | Term live birth, Apgar score 10, birth weight 3340g | Generally in good condition |
| 7* | VSD | CMA(VOUS) | Term live birth, small for gestational age, Apgar score 10, birth weight 2490g | Post-surgery, speech delay |
| 8* | VSD, fetal cleft lip and palate | CMA(VOUS) | Term live birth, Apgar score 10, birth weight 3800g | Generally in good condition |
| 9* | VSD, nasal bone hypoplasia | CMA(VOUS) | Term live birth, small for gestational age | Live birth, lost to follow-up |
| 10* | VSD | CMA(VOUS) | Term live birth, Apgar score 10, birth weight 3135g | Post-surgery, generally in good condition |
| 11* | VSD | CMA(VOUS) | Term live birth, Apgar score 10, birth weight 3405g | Generally in good condition |
| 12* | VSD | CMA(VOUS) | Term live birth, Apgar score 10, birth weight 3970g | Generally in good condition |
| 13* | VSD | CMA(VOUS) | Term live birth, Apgar score 10, birth weight 3010g | Generally in good condition |
| 14* | PLSVC | CMA(VOUS) | Term live birth, Apgar score 10, birth weight 3145g | Generally in good condition |
| 15* | PLSVC, possible right pelvic ectopic kidney | CMA(VOUS) | Term live birth, Apgar score 10, birth weight 2580g | Generally in good condition |
| 16* | PLSVC | CMA(VOUS) | Term live birth, Apgar score 10, birth weight 3005g | Generally in good condition |
| 17* | PLSVC | CMA(VOUS) | 35-week preterm birth, Apgar score 10, birth weight 1820g | Generally in good condition |
| 22* | Aberrant right subclavian artery | CMA(VOUS) | Term live birth, Apgar score 10, birth weight 3180g | Generally in good condition |
| 24* | Coarctation of the aorta, anomalous ductus venosus connection | CMA(VOUS) | Term live birth, Apgar score 8, birth weight 2230g | Post-surgery, generally in good condition |
| 25* | Aortic stenosis | CMA(VOUS) | Term live birth, Apgar score 10, birth weight 2875g | Live birth, lost to follow-up |
| 26* | Tricuspid stenosis | CMA(VOUS) | Term live birth, Apgar score 9, birth weight 3370g | Live birth, died at 2 months of age |
| 16c | TOF | WES(VOUS TRPM4) | Term live birth, Apgar score 9, birth weight 3200g | Post-surgery, generally in good condition |
| Note:*: Corresponds to the case number in Supplementary Table 2; c: Corresponds to the case number in Supplementary Table 3. | | | | |
